# The Role of Zinc and Copper in Gynecological Malignancies

**DOI:** 10.3390/nu12123732

**Published:** 2020-12-03

**Authors:** Kaja Michalczyk, Aneta Cymbaluk-Płoska

**Affiliations:** Department of Gynecological Surgery and Oncology of Adults and Adolescents, Pomeranian Medical University, al. Powstańców Wielkopolskich 72, 70-111 Szczecin, Poland; aneta.cymbaluk@gmail.com

**Keywords:** zinc, copper, micronutrients, metallothionein, gynecological cancer

## Abstract

Zinc (Zn) and copper (Cu) are essential microelements, which take part in cellular metabolism, feature in enzymatic systems, and regulate enzyme activity. Homeostasis of these micronutrients is tightly regulated by multiple compensatory mechanisms that balance their concentrations including transporters, importers, and metallothioneins. An altered intake of only one of these trace elements may cause an imbalance in their levels and result in their competition for absorption. Relatively low levels of zinc and increased levels of copper may result in an increased level of oxidative stress and impair the antioxidant properties of multiple enzymes. Altered levels of trace elements were discovered in various pathologies including immunological, degenerative, and inflammatory diseases. Moreover, due to the role of Zn and Cu in oxidative stress and chronic inflammation, they were found to influence cancerogenesis. We review the roles of zinc and copper and their mechanisms in tumor growth, metastasis potential, microenvironment remodeling, and drug resistance. We highlight their role as potential biomarkers for cancer diagnosis, treatment, and prognosis, concentrating on their impact on gynecological malignancies.

## 1. Introduction

Zinc and copper are important micronutrients traceable in different tissues. They are essential components of biological structures; however, in high concentrations, they may become toxic. They take part in cellular metabolism, feature in enzymatic systems, and influence enzyme activity. They have structural and storage functions, and they regulate gene expression and protein synthesis. Other transition metals that may be significant for human health and physiology are iron, manganese, selenium, cobalt, and chromium [1]. The levels of micronutrients may be altered by multiple environmental, dietary, or lifestyle factors including the impact of ambient air pollution and hazardous lifestyle habits. The action of zinc and copper has been demonstrated to have antioxidant and anti-inflammatory properties. Their amounts have been found to differ and to have clinical importance in age-related degenerative diseases, oxidative stress, inflammatory processes, and immunological diseases. Their levels were also correlated with patients’ nutritional status and age [2,3].

Zinc is one of the most important trace elements in the human body. It can act in three ways: as a catalyst, a structural ion, and a regulatory ion [4]. Zinc has a critical role in maintaining homeostasis, immune functions, and oxidative stress. It is also important in cell aging and apoptosis [4]. It acts as a cofactor to more than 300 enzymes; however, its key role is in the stabilization of protein structure including signaling enzymes [5,6]. Zinc is found in all body tissues and secretions, with the highest concentrations in muscles, bones, skin, and liver [4]. When compared with other transition metal ions, including copper and iron, zinc is the only one that does not undergo redox reactions as it has a filled *d* shell. Excessive dietary Zn can cause Cu deficiency [7].

Copper (Cu) is a necessary element for the development of connective tissue, nerves, and bones. In the organism, it is transported by ceruloplasmin. The highest concentrations of Cu are in the liver and the brain, with a total concentration of 80 mg of Cu in the adult body [1]. Cu deficiency in humans is rare; however, it can cause severe complications such as anemia, leucopenia, and osteoporosis.

Despite numerous studies, the exact role of trace metals in carcinogenesis is not yet clearly understood. There is a great interest in the role of trace elements in cancer etiology and the difference between their concentrations in malignant and normal tissues. This review summarizes the role of zinc and copper as multipurpose nutrients and their biological roles in homeostasis, proliferation, and apoptosis. We review their role and interdependence in oncogenesis and the formation of gynecological malignancies. For the purpose of our review, we conducted a literature search on Pubmed, Web of Science, and Cochrane Library databases including studies up to October 2020. We evaluated the information provided in articles published in English using a combination of keywords relevant to zinc, copper, cancer, and gynecological malignancies.

## 2. Zinc

Zinc is an important micronutrient and is required for cell proliferation, differentiation, growth, and development. It takes part in the maintenance of homeostasis, DNA synthesis, RNA transcription, cell division, and activation. It is also involved in immune responses, oxidative stress, apoptosis, and aging [8]. Its deficiency was found to increase the levels of lipid peroxidation in the mitochondrial and microsomal membrane and the osmotic fragility of the erythrocyte membrane.

In the human body, zinc is required for the proper function and wellbeing of the organism, and it varies in accordance with dietary habits, geographic localization, and climate. Moreover, the presence of any stress conditions including trauma, parasitic infestations, and infections may affect patients’ need for zinc intake. Diet and supplements are the main sources of zinc intake and account for approximately 90–95% of its intake. The general recommended daily dietary intake of Zn is estimated to be 15 mg/day [9]. However, as acute and/or chronic zinc poisoning may occur, levels of zinc should be controlled within a suitable range [10].

Due to the unique properties of zinc, its use may give therapeutic benefits in the treatment of diseases, in which a concurrent zinc deficiency can complicate the clinical features. It can affect in an adverse manner a patient’s immunological status, as well as cause an increase in oxidation stress and/or generation of inflammatory cytokines [4]. Both oxidative stress and chronic inflammation may increase the risk or cause the development of many chronic diseases (e.g., atherosclerosis, malignancies, neurological disorders, and autoimmune diseases) [11]. Zinc is necessary for major body processes such as blood clotting, proper thyroid function, cognitive function, bone mineralization, the functioning of the immune system, prostaglandin production, and wound healing [4].

Changes in zinc concentration can influence both adaptive and innate immunity, affecting the immune system’s mechanism of anticancer activity. It is important to assess the status of zinc in patients and correct zinc deficiency, since its properties may cause a significant impact on the effects of the therapy. Depleted levels of zinc have been associated with a reduction in monocyte adhesion to the endothelium, granulocyte chemotaxis, and macrophage phagocytosis. Lower zinc levels were also shown to decrease the cytotoxicity and activity of natural killer cells. Zinc depletion can also reduce the activity of cytokines secreted by T cells and macrophages, as well as T-cell differentiation [12]. Recent studies have shown that zinc could either protect against or cause cell apoptosis depending on its concentration. Moreover, the effect of exogenous zinc on the life and death of cells depends on the of intracellular levels of zinc present in the human body [13,14].

Multiple studies have tried to evaluate the role of trace elements in different types of cancer. The involvement of Zn in cancer development has been supported with abnormal levels of zinc in the serum and malignant tissue of examined patients with various types of malignancies [15,16,17].

### 2.1. Zinc Homeostasis

**Figure 1:**
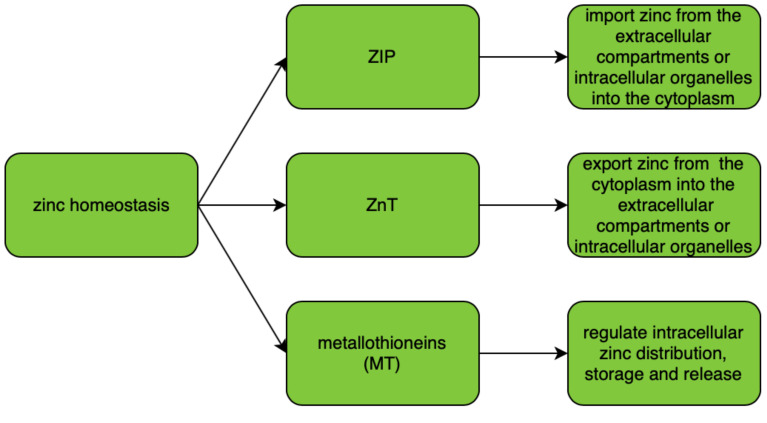
Regulation of zinc homeostasis.

ZIP (zinc importers), ZNT (zinc transporters), and zinc-sequestering metallothioneins (MT) are proteins that maintain intracellular zinc homeostasis. Their roles in zinc homeostasis are described in Figure 1. The homeostasis is tightly regulated and controlled by changes in zinc influx and outflux across cell membranes. As zinc particles cannot freely cross cellular membranes, they use specific zinc-permeable channels and transporters, of which ZNT and ZIP proteins are the most important [18]. The family of plasma membrane-localized zinc transporters (ZIP transporters, also called solute carrier 39A (SLC39A)) consists of 14 subtypes (ZIP1–14). They facilitate the influx of zinc into the cytosol either from extracellular fluid or from intracellular vesicles [19,20,21,22].

Intracellular zinc transport is mostly performed using ZNT transporters (also called SLC30A), which distribute zinc in the cytosol and cellular organelles. The ZNT family consists of 10 subtypes (ZNT1–10) of zinc transporters which lower intracellular zinc levels by mediating its efflux from the cell or influx into intracellular vesicles [20,22]. Malfunction of zinc transporters may result in zinc deficiency. There is increasing evidence that any dysregulation of and/or mutations in ZIP and ZNT transporter genes may cause functional disorders including diabetes and cancer [23]. A correlation between levels of expression of zinc transporters and their dysregulation or malfunction was found in various tumor types, suggesting that any change in the intracellular concentration of zinc and its homeostasis can contribute to the severity of the malignancy [24,25,26,27]. In various cancers, altered expression levels or abnormal activity of Zn transporters were noted; however, no specific mutations or variants of ZNT or ZIP were associated with a specific type of malignancy [18].

Both ZIP and ZNT transporters can be either tissue-specific or universally expressed in the body tissues depending on the subtype of the transporter [28]. Cellular zinc importers (ZIP) have been widely investigated and discovered to be upregulated in many patients presenting with different tumor types. This may be the reason why zinc concentration is increased in most tumor types. ZIP1, ZIP2, and ZIP3 have been found to be downregulated in the malignant cells of patients with prostate cancer [29]. Overexpression of ZIP4 was linked to increased cell proliferation of patients with pancreatic, prostate, lung, and ovarian cancer [18,24]. In patients with breast cancer, the expression of ZIP6 and ZIP10 was linked with metastasis to lymph nodes [30,31]. Increased levels of ZIP7 were correlated with increased tumor growth and invasion in tamoxifen-resistant breast cancer [32,33,34].

Alterations in intracellular homeostasis of zinc result in regulation of molecular targets such as protein kinase C (PKC), cyclic AMP (cAMP)-dependent protein kinase (PKA), Ca/calmodulin-dependent protein kinase II (CaMKII), phosphodiesterases (PDEs), protein tyrosine phosphatases (PTPs), and transcription factors (including nuclear factor (NF)-κB) [35].

### 2.2. Zinc and Metallothioneins

Zinc-binding proteins such as metallothioneins (MTs) are intracellular metal-binding proteins. They are present in all living organisms that mediate metal homeostasis. They also play an important role in protection against heavy metal toxicity, DNA damage, and oxidative stress. They are small cysteine-rich proteins.

There are four types of metallothioneins (MT-1, MT-2, MT-3, MT-4) that are encoded by the genes located on chromosome *16q13* [36]. MT-1 and MT-2 are the isoforms of metallothioneins which participate in zinc metabolism and its homeostasis. Zinc and copper are physiological inducers of metallothioneins [37]. Metallothioneins have an important role in the zinc effect on the immune system and are protective against stress and increase in aging [8]. Metallothioneins are crucial to the proper functioning of natural killer cells; their polymorphism can impair innate immunity [15]. An increased expression of MT isoforms has been demonstrated in some of the tumor cells.

The biological functions of metallothioneins are related to their high affinity for heavy metals; they can control cellular homeostasis of zinc and copper, essential for processes of cell proliferation, differentiation, and antioxidation. Moreover, they can act as antioxidants in order to protect cells against free radicals and mutagenic oxidative stress, antineoplastic drugs, and radiation [38,39]. MTs were found to play protective roles against DNA damage and apoptosis. Moreover, they were found to have important roles in cancerogenesis and cancer therapy as they participate in tumor growth, progression, metastasis, and drug resistance [36]. Metallothioneins can also bind other trace metals including copper, cadmium, mercury, and platinum.

Metallothioneins also influence the activity of Zn-dependent proteins including Cu/Zn superoxide dismutase (SOD), zinc finger proteins, transcription factors, and other proapoptotic proteins [40,41]. They were also found to take part in the processes required for angiogenesis. Multiple in vivo studies have reported an increased expression of MT-1 and MT-2 isoforms in tumor-associated angiogenesis; MTs may increase the synthesis and expression of fibroblast growth factors (FGFs), as well as other factors including transforming growth factor (TGF-β) and vascular endothelial growth factor (VEGF), resulting in the stimulation of tumor growth and better vascular supply [42].

### 2.3. Antioxidant Properties of Zinc

Oxidative stress is an important factor that contributes to the pathogenesis of chronic diseases, mutagenesis, cancerogenesis, atherosclerosis, vascular diseases, neurodegeneration, immunologic diseases, and aging processes. The role of zinc in cancerogenesis is mainly associated with its antioxidant properties, thanks to which it can potentially inhibit tumor growth. The intracellular redox potential system is a crucial element that ensures the maintenance of cellular homeostasis and regulation of various metabolic cell functions [43]. Under homeostasis, reactive oxygen species (ROS) act as mediators and regulate multiple cellular processes; they also induce cell differentiation, signaling, and apoptosis, as well as the release of nitric oxide (NO) and glucose transport. The role of the antioxidant system is to protect against the excessive concentration of ROS and their potential toxicity-related effects. The system consists of enzymatic proteins including superoxide dismutase (SOD1, SOD2), catalase (CAT), glutathione peroxidase (GPx), glutathione reductase (GR), peroxiredoxin (PRX), and nonenzymatic proteins, vitamins, and metal ions. Any alteration in the level of ROS can disturb the biological functions of the DNA, as well as other proteins and lipids, resulting in a permanent change in their structure and abnormalities in cellular metabolism.

One of the components of superoxide dismutase, which catalyzes the dismutation of superoxide radicals to hydrogen peroxide, is zinc. SOD is present in the human body as three isoenzymes. A reduction in its activity can cause oxidative stress and lead to neuron death, cancerogenesis, and/or tumor progression [44,45].

### 2.4. Zinc Finger

Zinc is a constituent of various metabolic enzymes, transcription factors, and cellular signaling proteins [8]. It is also the main structural component of zinc finger proteins (Zfp) and is required for their stability and stabilization. Zfp is the largest and most diverse group of nucleic acid-binding proteins, which play various important roles in the transcriptional regulation of cellular metabolic network. Zinc finger proteins are also responsible for the interaction with zinc-binding domains including zinc fingers, RING fingers, and LIM domains [46,47,48,49,50]. There are eight types of zinc fingers, which differ by their quaternary structure and the location of the amino acids which contain zinc molecules. Proteins containing zinc fingers (ZFs) have a selective ability to bind to DNA, RNA, and other proteins. Zinc finger transcription factors may directly influence tumor formation via the epithelial–mesenchymal transition [15].

Zinc finger domains are the major determinants of regulatory networks and processes as they are present in almost half of human transcription factors. Some ZFs have been demonstrated to mediate transcriptional repression through various interactions with chromatin-remodeling factors. As changes in chromatin were observed in cancers, ZFs may be of particular interest to be investigated in cancers [51,52]. Munro et al. [53] analyzed the somatic mutations found in *Cys2His2* zinc finger domains. They found that two of the functionally important specific positions within the zinc finger domains were mutated in patients with uterine corpus endometrial carcinoma, colon and rectal adenocarcinomas, and skin cutaneous melanoma.

A20 is one of the zinc-finger-transactivating proteins. It inhibits tumor necrosis factor (TNF)-α-induced NF-κB (nuclear factor kappa B) activation. Using this protein, zinc can inhibit NF-κB activation, which leads to a decrease in the generation of multiple inflammatory cytokines and adhesion molecules [54].

### 2.5. Matrix Metalloproteinases (MMPs)

Matrix metalloproteinases are a family of extracellular zinc-dependent endoproteinases that consist of more than 21 human metalloproteinases. MMPs can be divided into eight structural classes, and three of them are bound to the cellular membrane. MMPs are synthesized in an inactive form of zymogens that can be later activated by the proteinase cleavage. The activity of MMPs is regulated by various endogenous inhibitors (e.g., α2-macroglobulin, tissue inhibitors of metalloproteinases (TIMPs), small molecules with TIMP-like domains, and RECK (reversion-inducing cysteine-rich protein with Kazal motifs)). MMPs are known for their capacity for the degradation of extracellular matrix components [55]. The activity of matrix metalloproteinases is regulated through the function of tissue inhibitors of metalloproteinases (TIMPs).

Matrix metalloproteinases play important roles in tumor invasion, infiltration, metastasis, and tumor angiogenesis [56,57]. They were found to be upregulated in most cancer types, and their expression was often associated with poor patient survival. Several MMPs were found to be expressed by the cancer cells (e.g., MMP-7); however, others were synthesized by the tumor stromal cells (e.g., MMP-2, MMP-9) [55].

### 2.6. Zinc and Oncogenesis

Zinc has been found to have a role in oncogenesis. Its levels are directly affected by the cells present in the cancer microenvironment, including pro-inflammatory mast cells, which release high levels of zinc into the surrounding tissues [58]. The presence of mast cells in the tumor environment is thought to worsen patient prognosis. Moreover, their presence can cause changes in extracellular zinc levels and affect cellular response [15]. Many cytokines and growth factors including interleukin (IL)-6, hepatocyte growth factor, epidermal growth factor, and TNF-α are produced in the tumor microenvironment and affect the expression of multiple zinc transporters [59].

### 2.7. Zinc Supplementation

Improper dietary habits and imbalanced nutrition can cause dietary deficiency of many important micronutrients. It is estimated that approximately two billion people do not absorb an adequate amount of zinc from the diet and are at risk for deficiency of this trace metal. On the basis of epidemiological studies, an increased risk for cancer development has been established in cases of dietary zinc deficiency [60,61]. Taking into consideration the antioxidant properties of zinc, it is possible that its dietary deficiency can result in oxidative stress and molecular changes, thus increasing the risk of malignancy [62]. In a study by Lee et al. [63], it was demonstrated that the intake of dietary zinc was associated with a decreased risk of colon cancer. To our best knowledge, there were no studies assessing the impact of dietary supplementation of zinc and copper and the risk of gynecological malignancies. However, it should be noted that zinc supplementation during anticancer treatment, especially during chemotherapy, may be harmful and should be taken with special precaution under the doctors’ supervision. Supplements may interfere with the chemotherapeutic agents used as a part of the treatment [64].

## 3. Copper

Copper is another trace element; it is an essential nutrient for all species. The biological activity of copper, as of iron, manganese, and selenium, is highly associated with the presence of unpaired electrons, which allows them to participate in redox reactions. Copper imparts an effect on the activity of a range of enzymes important for cellular respiration, defense against free radicals, melanin synthesis, and formation of connective tissue. Changes in Cu levels can also affect iron metabolism. Examples of these enzymes include Cu/Zn superoxide dismutase (Cu/Zn SOD), ceruloplasmin, cytochrome oxidase, tyrosinase, dopamine hydroxylase, and lysine oxidase. Copper can also act as their cofactor and as their allosteric component.

Serum copper and ceruloplasmin levels are the commonly used parameters/indicators to assess the nutritional status of copper in the human body. The changes in serum copper level were found to vary depending on the sex of the patient (female vs. male) [65]. A high dietary intake of iron (Fe) and/or zinc (Zn) can adversely affect the concentration of Cu in the human body [66]. The recommended dietary allowance for copper for an adult is equal to 9 mg/day [67].

Copper levels are tightly regulated, and its imbalance can have implications on the development and progression of both chronic and inflammatory diseases (including metabolic, cardiovascular, and neurodegenerative diseases). It can also predispose to malignant processes [68]. A deficit in copper levels can cause impaired levels of energy, abnormal glucose and cholesterol metabolism, increased oxidative damage, and altered function and structure of circulating blood and immune cells [69,70]. Copper deficiency has been found to be associated with a higher frequency of infections [71], increased cardiovascular risk [66], and alterations in the metabolism of cholesterol [72,73].

In cases of copper deficiency, the oxidation system may be compromised. Cu is a catalytic cofactor for superoxide dismutase (Cu/Zn SOD) and ceruloplasmin. Its deficiency can also alter other enzymes including catalase and selenium-dependent glutathione peroxidase, as well as other reactive oxygen species (ROS) scavengers such as metallothioneins and glutathione [66]. Deregulation of oxidative stress impairs cell DNA repair mechanisms due to the overproduction of ROS and is an important mechanism for cancer development [74].

The role of copper and its impact on oncogenesis and cancer has been studied for several decades. Many reports have been published on the aberrant levels of copper in malignant tissues of tumor-bearing mice and cancer patients [75,76,77,78]. Elevated levels of copper have been found in the malignant tissues of breast, ovary, lung, and stomach. Copper is being investigated as a potential target for cancer treatment due to its elevated levels in malignant tissues and properties to promote angiogenesis, cancer growth, and metastasis. Copper chelation to decrease its bioavailability continues to be investigated among various clinical studies with the goal of causing the inhibition of angiogenesis in various types of cancer [79].

### 3.1. Ceruloplasmin

Ceruloplasmin is the main carrier of copper in the human body. It is an enzyme synthesized in the hepatocytes and secreted into plasma after the incorporation of copper atoms. Ceruloplasmin contains about 90% of serum copper. It also participates in iron homeostasis [80]. Ceruloplasmin is an acute phase protein; its synthesis and secretion can be significantly increased due to inflammatory processes, infection, diabetes, cardiovascular diseases, angiogenesis, pregnancy, and cancer [66]. Even though copper levels do not affect ceruloplasmin synthesis or secretion, its deficiency decreases ceruloplasmin activity as it fails to incorporate copper into its structure and decreases its stability [81].

Ceruloplasmin is an adipokine with an increased expression in the adipose tissue of obese patients, as well as in cells present in obesity-associated cancers [82]. Knekt et al. [83] conducted a study to evaluate the relationship between serum ceruloplasmin levels and cancer incidence. They noted that the overall incidence of cancer was positively associated with serum ceruloplasmin levels. The association was the strongest for patients diagnosed with lung cancer.

### 3.2. Copper Homeostasis

The copper homeostasis system is a complex system of networks of proteins that deliver Cu^+^ to Cu-dependent proteins. The network participates in cell protection against the harmful effects of excess Cu. In the cells of human body, copper is present in its intracellular form of Cu^+^, which is highly toxic (as it can react with molecular oxygen or hydrogen peroxide and produce free radicals). The main Cu transporter in the human cells is copper transporter 1 (CTR1), a protein built of 190 amino acids. The Cu^+^ ions enter the cells via CTR1 and are moved to pathway-specific chaperones (including antioxidant protein 1 (ATOX1), copper chaperone for superoxide dismutase (CCS), and the COX17 cytochrome c oxidase assembly homolog) that deliver copper to various organelles and enzymes that require its presence [84]. The expression of CTR1 is regulated by Cu concentrations. Elevated concentrations of Cu were found to trigger the endocytosis of CTR1 [85]. Other Cu transporters that take part in Cu uptake in mammalian and eukaryotic cells are CTR2 and DMT1 (divalent metal transporter 1) [86].

### 3.3. Copper and Metallothioneins

Metallothioneins are involved in zinc and copper homeostasis, storage, and protection against their toxicity. MTs act as antioxidants due to their high degree of cysteines, which are subject to sulfhydryl oxidation [87,88]. It was observed in rat models with Wilson’s disease that, under the conditions of significant Cu excess, metallothionein synthesis is increased [89]. Moreover, increased MT levels have been observed under conditions of oxidative stress and environmental toxicity. Metallothioneins were shown to be more effective than glutathione in the prevention of DNA damage induced by hydroxyl radicals [90].

### 3.4. Copper and Oxidation

Copper is a highly redox-active element and readily donates and accepts electrons that can be shifted between its two valence states. Many important enzymes harness this activity, which is why copper has important roles in redox reactions that occur in the organism [79]. Due to the ability of copper to produce excessive amounts of ROS, numerous transporters and Cu chaperones protect the cells and regulate the uptake of copper, as well as its distribution, efflux, and delivery [66]. Copper deficiency has been demonstrated to decrease Cu/Zn SOD activity and increase the number of superoxide anions in Cu-deficient rats [91]. Moreover, Johnson and Thomas [92] found that Cu deficiency causes a decrease in cytochrome c oxidase (COX) activity, and the oxidative inactivation of complex I (nicotinamide adenine dinucleotide (NADH) ubiquinone oxidoreductase) contributes to the increased production of ROS.

### 3.5. Copper and Angiogenesis

As discovered by Folkman [93], the growth, invasion, and metastasis of a tumor are dependent on angiogenesis; the tumors cannot grow any larger than 1 mm^3^ without the formation of new blood vessels [94]. Copper has proangiogenic properties and can induce the migration of endothelial cells at an early stage of angiogenesis. Copper seems to be a necessary element for endothelial cell activation as it stimulates their proliferation and migration. There are multiple and varied molecular pathways in which copper induces a proangiogenic response. For example, it can directly bind to the angiogenic growth factor. It can also enhance its affinity for endothelial cells [95,96]. It is the only transition metal found to be a cofactor required for several angiogenic mediators including vascular endothelial growth factor (VEGF), basic fibroblast growth factor (bFGF), interleukin 1 (IL-1), and IL-8 [97,98,99]. Moreover, copper is involved in the regulation of the secretion of angiogenic molecules such as fibroblast growth factors (FGFs) and IL-1α [100]. Due to the multiple roles of copper in the regulation of angiogenesis, it has the potential to be involved in tumor metastasis.

### 3.6. Copper and Chemoresistance

Chemoresistance is an important problem in the failure of cancer treatment. Despite multiple molecular mechanisms of chemoresistance, there is some evidence that copper transporters play a central role in drug resistance, particularly in the chemoresistance of platinum-based therapeutics. Platinum-based compounds such as carboplatin and cisplatin are antineoplastic agents commonly used in therapeutic protocols including gynecological malignancies such as ovarian and endometrial cancer. Changes in the expression, activity, and/or the cellular localization of copper transporters were linked with the presence of cancer cells, especially ovarian cancer and non-small-cell lung cancer, that developed resistance to platinum drugs such as cisplatin [79]. High-affinity copper transporter CTR1 can mediate the cellular uptake of platinum-based chemotherapeutics, including cisplatin. When the expression of CTR1 is depleted, many cell types were demonstrated to accumulate less platinum-based drugs and, therefore, were more resistant to these chemotherapeutics [101,102]. A meta-analysis by Sun et al. [103] showed that increased CTR1 expression was significantly associated with a favorable overall and disease-free survival, suggesting that CTR1 could be a potential prognostic factor for the survival of cancer patients who underwent chemotherapy. Moreover, it could become a treatment target to overcome platinum resistance.

## 4. Cu/Zn Ratio

The intra- and extracellular levels of both copper and zinc are tightly regulated by compensatory mechanisms, which aim at the stabilization of the trace-metal concentrations within proper ranges of nutritional intake. Their regulation mainly involves the transfer of zinc and copper from the intestinal lumen to the portal circulation through the absorptive cells [104]. However, the levels may vary on the basis of their dietary intake levels and the efficiency of absorption. High zinc intake was found to have the ability to decrease copper absorption. Moreover, other dietary components may promote or impair the absorption of these substances [105,106]. Cu and Zn are cofactors for a variety of enzymes. One of them is copper/zinc superoxide dismutase, which is a powerful intracellular enzyme participating in antioxidant defense and the removal of highly reactive superoxides. An altered intake of only one of these trace elements may cause alterations and an imbalance in their levels, resulting in their competition for absorption. Relatively low levels of zinc and increased levels of copper may result in increased oxidative stress and impair the antioxidant properties of multiple enzymes [105].

Several studies have shown higher circulating levels of copper, lower levels of zinc, and an altered ratio of these to micronutrients. An increased Cu/Zn ratio was noted in many different malignancies including digestive tract [107,108], gallbladder [109], lung [110], breast [111,112], ovarian [113,114], endometrial [115], and cervical cancer [116]. It was suggested that the Cu/Zn ratio could be potentially used as a clinical diagnosis of neoplasms of the digestive system [107,108]. Furthermore, a study on head, neck, and face tumors demonstrated the Cu/Zn ratio to be a reliable parameter. The Cu/Zn ratio was found to have diagnostic and prognostic properties. The alterations in Cu/Zn levels were related to the stage of the disease. Moreover, after the treatment, the ratios were either coming near to normal limits or were maintained at post-therapy levels [117]. The use of the Cu/Zn ratio should be further investigated to evaluate its possible role in various gynecological malignancies.

## 5. Cu, Zn, and Gynecological Malignancies

There is limited information on the levels of trace elements and their impact on the cancers of the female reproductive system. Due to an alteration of optimal levels of trace elements, oxidative stress may occur and cause metabolic disturbances and changes in the cellular structure. Increased exposure to reactive oxygen species (ROS) can cause damage and mutation of the DNA and lead to the initiation of carcinogenesis. The levels of trace metals and transporters involved in their homeostasis may serve as biomarkers in diagnostics processes, as well as the monitoring of chemotherapeutical treatment. Trace metals act as cofactors to multiple enzymes, and changes in their concentration can affect the formation of free radicals, tumor growth, and angiogenesis. Metallothioneins were found to participate in tumor growth, progression, metastasis, and drug resistance. Below, we describe the studies on these micronutrients, their rationale, and their potential future clinical use in gynecological malignancies.

A study by Michos et al. [118] discovered significant changes in copper and zinc levels in the plasma during the menstrual cycle in women. Alterations in the levels of copper during the various phases of the menstrual cycle correlated negatively with the changes in estradiol levels, whereas the changes in Zn correlated positively with those in estradiol. The authors concluded that the cyclic fluctuation of plasma levels of the studied trace metals may be due to the cyclic fluctuation of plasma levels of estradiol. The concentrations of circulating estrogens and progesterone were found to influence a series of blood indicators commonly used in the copper status assessment, including serum copper, ceruloplasmin, and aminotransferases [65]. Moreover, cellular studies demonstrated that estradiols increase copper transporters and, hence, cell copper uptake [119].

Gynecological malignancies are often hormone-related. The estrogen receptor can be activated by metalloestrogens and metalloids. Metalloestrogens can be grouped into two separate subclasses: oxyanions (including arsenite, antimony, nitrite, selenite, and vanadate) and bivalent cations (cadmium, calcium, cobalt, copper, nickel, chromium, lead, mercury, and tin) [120].

Many actions of the estrogens are mediated by the two isoforms of estrogen receptor, Erα and Erβ. Erα mediates the mitogenic actions associated with the hormone, while Erβ has an antimitogenic effect [121]. Copper was demonstrated to have a high affinity to the Erα receptor and to bind with it, blocking the binding of estradiol. Moreover, it was found to be able to interact with the same amino acids in the ligand blocking the domain [122]. Copper, cobalt, nickel, lead, mercury, tin, and chromium(II) were found to induce the proliferation of cells in estrogen-dependent breast cancer [122,123,124] and to increase the transcription and expression of estrogen-regulated genes [122].

### 5.1. Cervical Cancer

Cervical cancer is one of the most common gynecological malignancies and caused 265,700 deaths worldwide in 2012. It is still one of the leading causes of death in low/moderate-income countries [125]; however, it is rare in high-income countries due to screening programs that allow the detection and removal of precancerous lesions. Its prevalence also varies according to human papillomavirus (HPV) infection prevalence from as low as 5% in North America to 21% in Africa [125,126]. Several risk factors have been identified and linked to cervical cancer, including HPV and smoking.

Infection with the high-risk strains of HPV is considered to be the most important risk factor of cervical cancer. Research has addressed the influence of nutrition on the prevalence of HPV infection and its persistence. Furthermore, the progression of the disease was demonstrated to be influenced by the presence of HPV infection. Literature has reported that a low intake of folic acid is related to a high risk of cervical cancer and the persistence of HPV infection [127,128]. It has been suggested that zinc ions (Zn^2+^) may inhibit viral replication and be a cofactor in the viral functions of many viruses [129]. Zinc is a component of various viral enzymes, proteases, and polymerases, and the regulation of cellular and systemic zinc distribution is crucial in the inhibition of viral replication and dissemination [130].

Levels of various trace metals have been investigated in patients with cervical cancer. A meta-analysis by He et al. revealed that serum selenium exposure may be a protective factor for cervical cancer [131]. Depleted levels of serum folate were found to be associated with an increased risk of cervical cancer in Asian populations [127].

One of the most important factors in the pathogenesis of cervical cancer is the inflammation of the squamous epithelium and glandular epithelium causing cervicitis. Recurrent and chronic inflammation of the epithelium may lead to the metaplasia and infiltration of deepening levels of the involved epithelium, resulting in cervical cancer. Studies have reported elevated levels of lipid peroxides, decreased levels of nonenzymatic antioxidants, and decreased activities of antioxidant enzymes in patients with cervical cancer [128]. A study by Cunzhi et al. [132] found lower tissue concentrations of Zn in patients presenting with cervical cancer compared to patients with non-lesion tissue. Higher concentrations of copper and Cu/Zn ratio were noted among patients with cervical cancer. As reported in a meta-analysis conducted by Xie et al., serum zinc levels were significantly lower in patients diagnosed with cervical cancer than in the control group; therefore, higher serum zinc levels may be a protective factor for cervical cancer in Asian women [133]. Additionally, a meta-analysis on serum copper level was performed by Zhang et al. [134], who found significantly higher serum copper levels in patients with cervical cancer. Proteins responsible for the maintenance of zinc homeostasis may also play an important role in the pathogenesis of cervical cancer. ZIP7 (SLC39A7) is a zinc transporter that was found to influence the progression of cervical cancer. Phosphorylation of the conserved zinc residues in the ZIP7 result in tyrosine kinase activation, protein kinase B (Akt) phosphorylation, and extracellular signal-regulated kinase (ERK) 1/2 signaling pathway activation [135]. The silencing of ZIP7 could be used as a potential therapeutic target for cervical cancer treatment [136].

### 5.2. Endometrial Cancer

Endometrial cancer is the second most common gynecological malignancy and its incidence is increasing, especially in moderate/high-socioeconomic countries. A number of factors have been identified to increase the prevalence of endometrial cancer including obesity, advancing age, late menopause onset, lower age of menarche, chronic anovulation, polycystic ovarian syndrome (PCOS), nulliparity, estrogen therapy in the absence of progesterone, therapy with tamoxifen, and Lynch syndrome [137,138]. The prevalence of endometrial cancer is highest in patients aged above 60 years old. Although most endometrial cancers are diagnosed at an early stage and are confined to the uterus, others can spread through the myometrial invasion and metastasize to different distant sites including the lymph nodes, liver, and lung. The treatment of endometrial cancer, especially diagnosed at an advanced stage (FIGO (Fédération Internationale de Gynécologie et d’Obstétrique) stage III and IV), is often problematic. Despite the use of multimodal treatment involving extensive surgery and chemotherapy, patients often face cancer recurrence. Up to date, no specific serum markers have been established for clinical use in the diagnosis and treatment of patients with endometrial cancer. Cu, Zn, and their ratio have been demonstrated to have clinical importance in age-related degenerative, diseases, oxidative stress, and inflammation, which may affect carcinogenesis. However, there is limited research concerning the importance of trace elements in endometrial cancer.

Atakul et al. [115] evaluated serum concentrations of Cu and Zn in relation to the metabolic profile and clinicopathologic features of patients suffering from endometrial cancer. They found lower levels of Cu, Zn, and Cu/Zn ratio in sera of patients with endometrial cancer when compared to the control group. Patients with greater myometrial invasion presented with lower levels of Cu compared to the group of patients with a lower degree with myometrial invasion. Patient age, body mass index (BMI), and gravidity were found to have no impact on zinc levels. Furthermore, a study on trace metals in endometrial cancer by Yaman et al. [139] reported similar levels of Cu and lower levels of Zn in cancer tissue of patients with endometrial cancer. Rzymski et al. [140] demonstrated elevated levels of Cu and Cu/Zn; however, they did not see any alterations in Zn levels. They also found decreased levels of Cu in postmenopausal patients with endometrial and endocervical polyps and reported that the levels of Cu might have been generally decreased due to a decreased Cu status in patients due to the cessation of postmenopausal estrogen.

Advanced age has been found as predisposing to increased oxidation and higher Cu/Zn ratios. Despite the fact that endometrial cancer usually affects patients of postmenopausal age, Atakul et al. [115] noticed lower levels of Cu/Zn ratios in their study on patients with endometrial cancer. Further studies are required to determine the clinical relevance of Cu/Zn ratio.

Pejic et al. investigated changes in activities and levels of copper/zinc superoxide dismutase (Cu-Zn SOD) and lipid hydroperoxides (LOOH) in the blood and endometrial tissue of patients with benign, hyperplastic, and malignant endometrium [141]. They found decreased SOD activity and level, as well as increased LOOH level, in all examined patients in comparison to healthy subjects and suggested that these patients may be more susceptible to oxidative damage caused by reactive oxygen species (ROS).

A study by Bidus et al. [142] evaluated the gene expression profiles of patient tissues with endometrioid endometrial cancers associated with lymph node metastasis in an effort to identify genes associated with metastatic spread. They found that the ZIC2 zinc finger gene was overexpressed in endometrial cancers with positive nodes when compared to those with negative nodes.

Our center was the first to investigate the differences between the serum levels of metalloproteinase-2 in patients with endometrial cancer and normal endometrium. Cymbaluk-Płoska et al. [143] observed that the levels of MMP-2 in endometrial cancer patients were significantly higher when compared to the control group of patients with normal endometrium. The sensitivity and specificity of the protein in the detection of endometrial cancer were noted as equal to 68% and 86%, respectively. Liu et al. [144] performed a systematic review and meta-analysis on the clinical significance of MMP-2 in endometrial cancer. Their results indicated that MMP-2 was expressed in a high percentage of tissues of patients with endometrial cancer and that its expression may be closely associated with the clinical stage, tumor invasion, and metastasis. They concluded that an indication of MMP-2 overexpression may be a predictive factor for the poor prognosis of patients diagnosed with endometrial cancer.

Moreover, the clinical relevance of the NGAL (neutrophil gelatinase-associated lipocalin)/MMP-9 pathway was investigated in patients with endometrial cancer. The authors discovered higher levels of NGAL and MMP-9 in patients with endometrial cancer when compared to the control group of patients with healthy endometrium and benign endometrial lesions, and they concluded that the use of NGAL/MMP-9 complex may be useful in the assessment of tumor stage before the surgical treatment [145]. Karahan et al. also observed an increased expression of MMP-9, MMP-2, and cyclooxygenase 2 (COX-2) in patients with primary endometrial carcinomas [146]. The abovementioned results show that metalloproteins, especially MMP-2 and MMP-9, are promising serum markers that could be potentially used in the diagnostic and therapeutic processes of endometrial cancer. Further research on larger cohort groups is required to identify the relevance of these findings and their possible use.

### 5.3. Ovarian Cancer

Ovarian cancer continues to be the most lethal cancer among gynecological malignancies. It is characterized by a rapid onset and poor prognosis. The therapies that are currently used in ovarian cancer treatment are limited by a high frequency of drug-resistant recurrent tumors, high rates of relapses, and low 5-year survival rates. Early detection and effective treatment for ovarian cancer are crucial to improving patient survival. Etiologic factors for ovarian cancers are not yet fully established. In addition to genetic and reproductive risk factors, chronic inflammation, oxidative stress, and free radical damage to the epithelial cells have been investigated and hypothesized to have a role in ovarian carcinogenesis [147]. It is still unclear how zinc and copper impact ovarian malignancy.

Yaman et al. [139] reported higher copper and similar zinc levels in tissues of patients with ovarian cancer. The mechanism of the elevation of Cu levels in malignant ovarian tissues may be explained by alterations and modifications in the relationship among trace elements with reduced catabolism or increased neoplastic synthesis of ceruloplasmin [139]. A recent meta-analysis by Lin et al. [148] demonstrated increased circulating copper and decreased zinc concentrations in patients diagnosed with ovarian cancer. The Cu/Zn ratio has been proposed as a marker of ovarian carcinoma. Gal et al. [149] tried using the copper-to-zinc ratio in addition to standard marker CA125 (carcinoma antigen 125) in patients undergoing exploratory surgery for suspicion of ovarian cancer. The model was found to be sensitive in the prediction of ovarian cancer before laparotomy. Moreover, Nayak et al. [150] found significantly increased levels of copper and ceruloplasmin levels in patients with ovarian cancer. They also noticed an increase in serum copper/ceruloplasmin ratio.

Proteins responsible for zinc homeostasis were also demonstrated to impact the formation of ovarian cancer and its metastasis. ZIP4 is one of the zinc transporters. It stimulates cell proliferation, cellular activities, drug resistance, and tumor progression. ZIP4 was found to have a role in the tumor promotion of many cancer types including pancreatic cancer, breast cancer, hepatocellular carcinoma, and glioma [151,152]. A study by Fan et al. [153] demonstrated ZIP4 overexpression in epithelial ovarian cancer tissues. It would be interesting to see if other zinc importers and/or transporters are overexpressed in malignant tissues of patients diagnosed with ovarian cancer or other gynecological malignancies and if they correlate with clinical staging.

Additionally, matrix metalloproteinases are known to have an important role in cancer cell invasion as they mediate the degradation of extracellular matrix proteins. The expression of matrix metalloproteinase-2 (MMP-2) has been linked to invasive metastasis in various neoplasia including ovarian, breast, and colon carcinoma. The expression of MMP-2 protein or messenger RNA (mRNA) was found to predict a poor prognosis. A study by Davidson et al. found that MMP-2 and MMP-9 were valid markers of poor survival in advanced-stage ovarian carcinoma [154]. Sakata et al. [155] suggested that the overexpression of MMP-2, MT1-MMP, TIMP-2, and MMP-9 and downregulation of TIMP-1 may contribute to the development or enhanced growth capacity of ovarian tumors. Moreover, they found that co-expression of MMP-2, MT1-MMP, and TIMP-2 within the same tumor seems to have an important role in ovarian cancer progression. They also hypothesized that elevated MMP-9 expression together with low expression of TIMP-1 may contribute to the lymph node metastasis of ovarian carcinoma cells. Another metalloproteinase that was investigated in ovarian cancer was MMP3. Significantly higher mean levels of serum MMP3 were noticed in patients with higher clinical staging compared to patients diagnosed with a lower staging. Moreover, the study demonstrated that baseline MMP3 concentrations were associated with patient survival and disease-free time [156].

A study by Zhang et al. [157] on ovarian cancer cell lines revealed that zinc contributes to ovarian tumor metastasis by promoting epithelial-to-mesenchymal transition (EMT) through a metal response transcriptional factor-1 (MTF-1)-dependent pathway. Zinc depletion by TPEN (N,N,N,N-Tetrakis (2-pyridylmethyl)-ethylenediamine), a membrane-permeable selective zinc chelator, may be a novel approach for ovarian cancer therapy by inhibiting EMT and attenuating the ERK1/2 and Akt pathways.

Ovarian cancer is usually detected at later stages of the disease, when the cancer cells have already spread into the abdominal cavity. It is the leading cause of death among gynecological malignancies, and there is an urgent need to develop novel therapeutic options to treat patients in the advanced stages. Zinc is an essential component of zinc finger transcription factors, which are targets of ovarian cancer therapies [158]. Haydee et al. targeted serous epithelial ovarian cancer cells with designer zinc finger transcription factors. They used a sequence-specific zinc finger artificial transcription factor (ATF) to upregulate the Maspin promoter in aggressive ovarian cancer cell lines. The systemic delivery of encapsulated particles resulted in inhibition of ovarian cancer cell growth in nude mice and showed potential for further clinical application for metastatic ovarian cancers.

Another proposed method to aid ovarian cancer treatment was to use metal oxide nanoparticles, including zinc oxide nanoparticles (ZnO-NPs), which show promise as anticancer agents. In research on cell lines by Padmanabhan et al. [159], ZnO-NPs were found to exhibit toxicity toward ovarian cancer cells, independent of the *p53* mutation status of the malignant cells of patients with ovarian cancer. ZnO-NPs induced oxidative and proteotoxic stress in ovarian cancer cells and caused cancer cell death via apoptosis. A different experiment on cell lines by Bae et al. [160] demonstrated the possibility of accumulation and uptake of zinc from exogenous supplementation. Bae et al. supplemented OVCAR-3 (ovarian carcinoma cell line 3) cells with zinc chloride and citrate (CIZAR^®^, (BH Biomedic Co., Seoul, Korea)), which resulted in a time- and dose-dependent decreased viability in ovarian cancer cells after administration of CIZAR^®^. Chemoresistance is an important issue in ovarian cancer treatment. Innovative research is required to develop methods that will allow either local or target chemotherapy to lower systematic chemotoxicity while providing a maximal dose of the chemotherapeutics to the malignant cells.

## 6. Conclusions

Zinc and copper seem to play an important role in oncogenesis due to their role in inflammatory and antioxidation processes. It is important that we continue the research on the pathogenesis of different malignancies. Gynecological cancers are one of the most frequent causes of death among females, and their etiology is yet not clearly understood. An imbalanced level of micronutrients in the human organism may cause alterations to many important processes including cell growth, division, and apoptosis. The maintenance of homeostasis of trace metals represents a potential way via which to reduce the chances of cancerogenesis. Metallothioneins are proteins that could be potentially used in the diagnostic processes, as well as prognosis of various malignancies, including gynecological cancers. Further studies including randomized controlled trials with consistency in doses and duration are needed. Micronutrients, especially copper and its transporters, may have a potential role in cancer treatment and can help to lower chemoresistance.
